# Antibacterial and antioxidant activity of methanol extract of *Evolvulus nummularius*

**DOI:** 10.4103/0253-7613.58514

**Published:** 2009-10

**Authors:** P.S. Pavithra, N. Sreevidya, Rama S. Verma

**Affiliations:** Department of Biotechnology, Indian Institute of Technology Madras, Chennai - 600 036, TN, India; 1 Sophisticated Analytical Instrumentation Facility, Indian Institute of Technology Madras, Chennai - 600 036, TN, India

**Keywords:** Antibacterial, DPPH, *Evolvulus nummularius*

## Abstract

**Objective:**

To evaluate the antibacterial and antioxidant activity of methanol extract of *Evolvulus nummularius* (L) L.

**Materials and Methods:**

Disc diffusion and broth serial dilution tests were used to determine the antibacterial activity of the methanol extract against two Gram-positive bacterial strains (*Bacillus subtilus* NCIM 2718, *Staphylococcus aureus* ATCC 25923) and three Gram-negative bacterial strains (*Pseudomonas aeruginosa* ATCC 27853, *Klebsiella pneumoniae* ATCC 70063 and *Escherichia coli* ATCC 25922). The methanol extract was subjected to preliminary phytochemical analysis. Free radical scavenging activity of the methanol extract at different concentrations was determined with 2, 2-diphenyl-1picrylhydrazyl (DPPH).

**Results:**

The susceptible organisms to the methanol extract were *Escherichia coli* (MIC=12.50 mg/ml) and *Bacillus subtilus* (MIC=3.125 mg/ml) and the most resistant strains were *Staphylococcus aureus, Klebsiella pneumoniae* and *Pseudomonas aeruginosa*. The methanol extracts exhibited radical scavenging activity with IC50 of 350 μg/ml.

**Conclusion:**

The results from the study show that methanol extract of *E.nummularius* has antibacterial activity. The antioxidant activity may be attributed to the presence of tannins, flavonoids and triterpenoids in the methanol extract. The antibacterial and antioxidant activity exhibited by the methanol extract can be corroborated to the usage of this plant in Indian folk medicine.

*Evolvulus nummularius* (L). L known as *Convolvulus nummularius* belongs to the family *Convolvulaceae* (Morning-glory). *E. nummularius* is a low growing species with creeping stem and rounded leaves. This herb is called by various names viz., Aakhukarni, Muusaakarni and Chhinipatra[1] and is widely distributed in India, Nepal, Bhutan, tropical America and Africa. In Indian traditional folk medicine, the whole plant is used as a medicine for hysteria, to cure burns, cuts, wounds and scropion stings.[2] In Nepal, the paste of the plant is used to treat scabies.[3] *E. nummularius* has been pharmacologically reported to possess antihelminthic activity,[4] wound healing activity,[5] poor sedative and anticonvulsant properties.[6] Three new compounds, 1-3 along with β-sitosterol and its glucoside, stigmasterol, *d*-mannitol, ursolic acid and oleanolic acid have been isolated from the aerial parts of *E. nummularius.*[7] However, the antibacterial and antioxidant activities of the methanol extract of *E. nummularius* have not been reported.

*E. nummularius* is a native species of Indian Institute of Technology Madras (IITM) campus. The forest of IITM has a rich biodiversity of flora and fauna. The forest has 298 species of plants. In an attempt to screen plants growing in the forest of our campus for medicinal properties, we studied the antibacterial and antioxidant properties of methanol extract of *E. nummularius*.

## Materials and Methods

### Plant material

*E. nummularius* (whole plant) were collected from the forest of Indian Institute of Technology campus, Guindy, Chennai for the study. The plant material was identified and authenticated by plant taxonomist, Dr. R.L.S. Sikarwar, Deendayal Research Institute, Chitrakoot.

### Preparation of methanol extract

The 200 g plant material was washed and air-dried in the shade for 15 days. The dried plant material was ground to a fine powder in a blender. A measured quantity of 20 g of dried powder was soaked in 200 ml methanol in round bottom flask at room temperature for 24 h. The extract was filtered with Whatman No.1 filter paper. The filtrate was allowed to dry at room temperature until dry methanol extract was obtained. The weight of the dried extract was 0.63 g. The yield of extract obtained was 3.15% (w/w). The extracts were stored in airtight containers at 4°C for further testing.

### Microorganisms

The antibacterial activity of methanol extract was determined by individually testing on Gram-positive and Gram-negative bacterial strains. The strains were obtained from NCIM (National Collection of Industrial Microorganisms, National Chemical Laboratory), Pune. The Gram-positive strains used were *Bacillus subtilus* (NCIM 2718) and *Staphylococcus aureus* (ATCC 25923). Gram-negative strains used were *Pseudomonas aeruginosa* (ATCC 27853), *Klebsiella pneumoniae* (ATCC 70063) and *Escherichia coli* (ATCC 25922). All the strains were maintained on nutrient agar at 4°C and were subcultured every month.

### Preliminary phytochemical tests

To detect the presence of possible phytochemicals in the methanol extract, preliminary phytochemical tests[8] were performed. (1) Test for alkaloids (1 ml extract + 1% hydrochloric acid on steam bath, 1 ml filtrate + 6 drops of Mayer's reagent, appearance of cream white precipitate indicated the presence of alkaloids. (2) Test for tannins (1 ml extract + few drops of 10% lead acetate), appearance of precipitate indicated the presence of tannins. (3) Test for saponins (1 ml of extract + 9 ml distilled water, shaken vigorously), appearance of stable froth indicated the presence of saponins. (4) Test for steroids and triterpenoids (Liebermann-Burchard) (2 ml extract + 1 ml chloroform + few drops of acetic anhydride + conc. sulfuric acid added along the side of test tube), appearance of blue or green color indicated the presence of steroids, and appearance of red, brown color indicates the presence of triterpenoids. (5) Test for cardiac glycosides (1ml extract + few drops of acetic acid + few drops of ferric chloride + 3-4 drops of conc. sulfuric acid), appearance of blue-green color indicated the presence of glycosides. Test for flavonoids (2 ml extract + conc. hydrochloric acid + magnesium ribbon), appearance of pink-red color indicated the presence of flavonoids.

### Antibacterial activity assay

The antibacterial activity of methanol extract was determined by disc diffusion and broth dilution method.[9] Nutrient Agar (NA) and Muller Hinton broth (MHB) were used for the tests. Overnight cultures were grown at 37°C in MHB. Bacterial suspensions of 1.0 × 10^8^ colony-forming units (CFU) per ml were obtained (OD_600_ = 0.08 nm). Petri plates containing 20 ml of NA were used for the disc diffusion assay. A total of 200 μl of the bacterial culture (10^8^ CFU) was spread over the surface of the plate and was allowed to dry for 10 min. The filter paper discs (6 mm in diameter) were loaded with methanol extract (3 mg/disc) and were allowed to dry completely. Discs with 10 μl DMSO and gentamicin (10 μg/disc) were placed as controls. The plates were incubated overnight at 37°C. The antibacterial activity against each test organism was quantified by determining average diameter of the zone of inhibition around the paper discs in millimeters. The tests were performed twice and average diameters of zones were calculated.

#### Determination of minimum inhibitory concentration (MIC) and minimum bactericidal concentration (MBC)

MIC of methanol extract was determined by serial dilution method. A total of 500 μl of MHB was added to tubes. Stock solution of 50 mg/ml of methanol extract was subjected to two fold dilutions such that concentration ranged between 50 mg/ml and 0.0156 mg/ml. Again, 10 μl of 10^6^ CFU bacterial suspensions were added to the tubes. The tubes were incubated at 37°C for 24 h. MIC was taken as the highest dilution of the extract that inhibited the growth of the bacteria. Lowest concentrations of the methanol extract, which inhibited the bacterial growth after a period of 24 h of incubation at 37°C, were recorded as MIC. Minimum bactericidal concentration (MBC) was determined by sub culturing 10 μl of the MIC tube solution (showing no visible growth) on a fresh drug free MHA plate and incubating for 24 h at 37°C. The highest dilution that yielded no bacterial growth was taken as MBC.[9]

#### Antioxidant assay

1,1 diphenyl-2-picryl hydrazy assay

The free radical scavenging activity of the methanol extract was measured with stable 1,1 diphenyl-2-picryl hydrazyl radical (DPPH) spectrophotometrically.[10] 0.004% DPPH solution was prepared in methanol. Test solutions of methanol extract were prepared in different concentrations (1 to 9 mg/ml). The absorbance was read at 517 nm. About 50 μl of extracts of different concentration was added to 2.950 ml of DPPH solution taken in a cuvette. The readings were measured at 517 nm for every 5 min interval for a total duration of 30 min. The scavenging activity was observed by bleaching of DPPH solution from violet color to light yellow. Ascorbic acid was used as control and 50 μl methanol was used as blank. The DPPH radical scavenging activity was calculated in terms of percentage inhibition using the formula, *% Inhibition = [100 (Ac-As)] / Ac* where *Ac* is the absorbance of the blank and *As* is the absorbance of the sample. The percentage of inhibition was calculated for ascorbic acid and different concentrations of methanol extract for a time interval of 30 min in step of 5 min. Each test was carried out twice. The tests for determining IC_50_ were repeated thrice, which showed a standard deviation of ±0.67.

### Results

**Figure 1:**
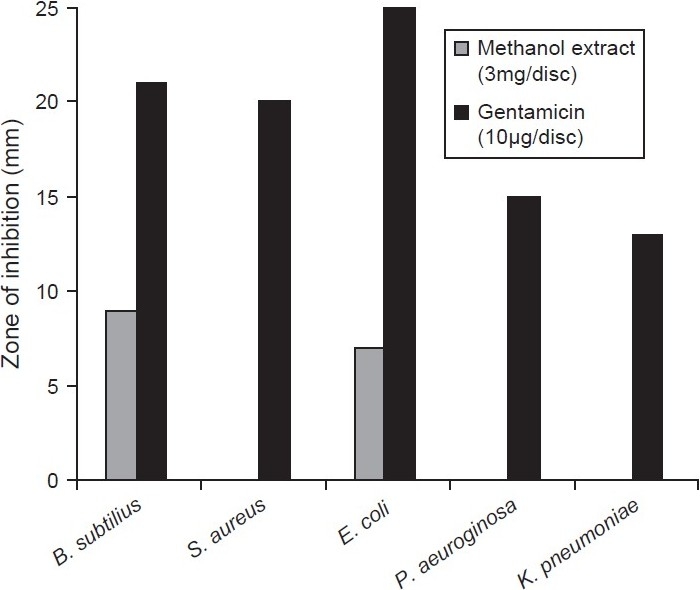
Antibacterial activity of methanol extract of *E. nummularius*: Comparisons of antibacterial activities of methanol extract of *E. nummularius* were performed along with the standard antibiotic gentamicin. Zone of inhibition was measured after 24 h incubation for methanol extract, gentamicin and plotted against bacterial strains

**Figure 2:**
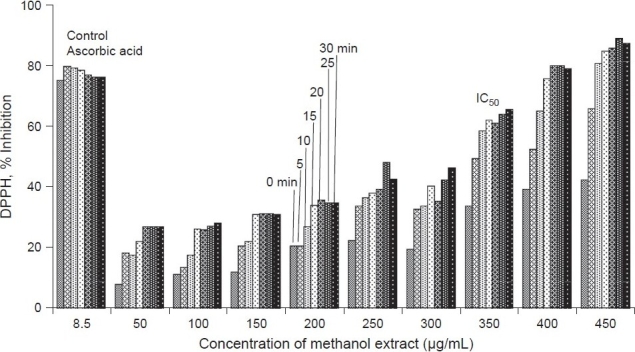
Free radical scavenging activity of methanol extract of *E. nummularius*: Free radical scavenging activities of ascorbic acid (control) and methanol extract of *E. nummularius* was done. The DPPH activities were measured for different concentrations of methanol extract at different time intervals. The results of each incubation were independently calculated and plotted as percent inhibition of DPPH against different concentrations of methanol extract

The phytochemical analysis of the methanol extracts performed in the present study showed the presence of alkaloids, triterpenoids, tannins, cardiac glycosides and flavonoids. The antibacterial activity of methanol extract of *E. nummularius* assayed by disc diffusion method is not comparable with the standard antibiotic gentamicin [Figure 1]. The MIC values for *E. coli* and *B. subtilus* were found to be 12.5 mg/ml and 3.125 mg/ml, respectively. Higher concentration of methanol extracts was needed for bactericidal action: The MBC values for *E. coli and B. subtilus* were found to be 25 mg/ml and 50 mg/ml, respectively. The methanol extract of this plant exhibited scavenging activity with IC_50_ = 350 μg/ ml. Figure 2 shows the increase in scavenging activity of the methanol extract with time at different concentrations. The scavenging activity was seen to increase gradually with increase in concentration of methanol extract. However, the scavenging activity was low in comparison with known scavenging substance, ascorbic acid.

### Discussion

In the present study, crude methanol extract isolated from *E. nummularius* exhibited both antibacterial and antioxidant activities. The study revealed that methanol extracts of *E. nummularius* is bactericidal against *E. coli* and *B. subtilus.* Presence of triterpenoids, polyphenols such as tannins and flavonoids in the methanol extract were detected in this study. The effect of antioxidants molecules on DPPH is due to their hydrogen donating ability.[11] The study shows that the extract has antiradical action and serves as free radical inhibitor or scavenger. Fenglina and co-workers reported that 56 plant extracts exhibited good scavenging activity out of 300 selected Chinese medicinal plants.[12] These extracts contained tannins, flavonoids and these plants have been used traditionally in the treatment of bleeding, dysentery, wounds and skin infections. Thus, the presence of above phytochemicals corroborate the reported scavenging activity exhibited by the methanol extract of *E. nummularius*, and supports its use in the treatment of wounds and burns as described in traditional medicine. These phytochemicals are known to have various pharmacological activities also.

Tannins are known for their astringent property and antimicrobial activity.[13] It is explained that in the wound healing process, the tannins bind to proteins of exposed tissues, thus precipitating the proteins, and forms antiseptic protective coat enabling the regeneration of new tissues to take place.[14,15] It is well known that tannins and flavonoids are also responsible for the strong free radical scavenging activity and anti-inflammatory property.[16,17] Free radical scavengers can inhibit the process of inflammatory response.[17] In a recent review article, cellular mechanisms for anti-inflammatory activity of flavonoids have been explained.[18] Flavonoids possess antioxidative, radical scavenging activities and regulate cellular activities of the inflammation-related cells: Mast cells, macrophages, lymphocytes, and neutrophils. For instance, some flavonoids inhibit histamine release from mast cells and others inhibit *t*-cell proliferation. In addition, certain flavonoids modulate metabolizing enzymes such as phospholipase A2 (PLA2), cyclooxygenase (COX), lipoxygenase (LOX) and the nitric oxide (NO) producing enzyme, nitric oxide synthase (NOS). An inhibition of these enzymes by flavonoids reduces the production of prostaglandins (PG), leukotrienes (LT), and NO, crucial mediators of inflammation. Thus, the inhibition of these enzymes by flavonoids is definitely one of the important cellular mechanisms of antiinflammation.[18] Triterpenoids also contain antiinflammatory, anticancer and antioxidant activities.[19] They are well known to promote the wound-healing process mainly due to their astringent and antimicrobial property, which seems to be responsible for wound contraction and increased rate of epithelialization.[20] Thus, the results of present work support the traditional use of *E. nummularius*.
